# Enzyme–Support Interactions of *Burkholderia cepacia* Lipase Immobilized on Silica Using Molecular Docking and Multitechnique Characterization

**DOI:** 10.1021/acsami.5c11931

**Published:** 2025-09-15

**Authors:** César A. Rodrigues, Jefferson C. B. Santos, Milson S. Barbosa, Nayára B. Carvalho, Meirielly S. Jesus, Ranyere L. Souza, Álvaro S. Lima, Matheus M. Pereira, Cleide M. F. Soares

**Affiliations:** † 67896 Universidade Tiradentes, Av. Murilo Dantas 300, Farolândia, Aracaju, Sergipe 49032-490, Brazil; ‡ Instituto de Tecnologia e Pesquisa, Av. Murilo Dantas 300, Prédio do ITP, Farolândia, Aracaju, Sergipe 49032-490, Brazil; § Instituto Federal de Educação, Ciência e Tecnologia de Minas Gerais (IFMG)—Campus Governador Valadares, Governador Valadares, Minas Gerais 35057-760, Brazil; ∥ CISAS—Center for Research and Development in Agrifood Systems and Sustainability, 112031 Instituto Politécnico de Viana do Castelo, Rua da Escola Industrial e Comercial Nun’Alvares 34, Viana do Castelo 4900-347, Portugal; ⊥ 37829 University of Coimbra, CERES, Department of Chemical Engineering, Rua Sílvio Lima, Pólo II—Pinal de Marrocos, Coimbra 3030-760, Portugal; # Departamento de Engenharia Química, UFBA, Universidade Federal da Bahia, Rua Aristides Novis 2, Federação, Salvador, Bahia 40170-115, Brazil

**Keywords:** nanoparticles, silica, NMR, sol−gel, simulations, esters

## Abstract

The influence of the microenvironment within enzyme–support complexes on the activity of *Burkholderia cepacia* lipase (BCL) immobilized on silica (SiO_2_–BCL) remains insufficiently understood. Without data on these interactions, it is challenging to determine whether modifications to the support material or immobilization protocols are needed for optimal enzyme activity and stability. Besides evaluating catalytic performance in esterification reactions, enzyme–support interactions were characterized using ^29^Si NMR, FTIR, BET, DSC, TGA, SEM–EDS, and molecular docking simulations. In this study, NMR^29^Si-based structural analysis revealed significant protein–surface (pore wall) interaction networks. The immobilization resulted in 88.6% efficiency and a protein loading of 17.72 mg·g^–1^, enabling further structural and functional characterization. Molecular docking elucidated the interaction mechanisms between silica functional groups (Q*^n^* sites) and BCL binding residues. Docking simulations indicated that the Q3 group interacts with the catalytic residue Ser87, potentially hindering substrate access to the active site. Spectroscopic and morphological analyses confirmed this interaction and correlated it with a significant decrease in enzymatic activity. Experimental evidence and FTIR analyses demonstrated that the increase in the α-helix content of SiO_2_–BCL correlated with the observed decrease in catalytic productivity. BCL exhibited a significantly higher esterification productivity (150.82 μmol·h^–1^·mg^–1^) than SiO_2_–BCL (28.10 μmol·h^–1^·mg^–1^), confirming the importance of optimal enzyme conformation for catalytic efficiency. The results highlight that, beyond the support’s composition, the spatial orientation and specific interactions with functional groups are critical determinants of catalytic efficiency. This integrative approach may guide the rational design of enzyme-based biocatalysts using mesoporous materials.

## Graphical abstract

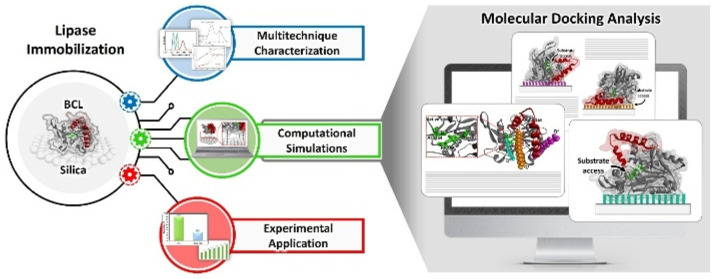

## Introduction

Immobilized lipases have gained increasing attention as alternatives to free enzymes, primarily due to their ability to retain enzymatic activity postimmobilization. However, understanding the underlying causes of specific activity variations remains challenging due to the numerous contributing factors.^1^ This lack of knowledge hinders the design and understanding at the molecular level of the bottlenecks in the immobilization process.^2^ Therefore, understanding the microenvironment associated with the support-enzyme interaction (adsorption of lipases to Si–OH groups in silica matrices) and their properties are of fundamental importance for the possible applications of the biocatalyst obtained in the immobilization process.^3^

Among the numerous applications of silica (SiO_2_), its role as a prominent support material stands out, particularly when synthesized via the sol–gel technique, which can be done via the sol–gel technique, one of the most commonly used techniques for immobilizing biomolecules and other chemical catalysts.^4^ Shuai et al.^5^ have described essential aspects of lipase immobilization, including the properties of the support and suitable reaction conditions.

The support structure must also have sufficient mechanical strength and resistance to chemical attack and microbial degradation.^6^ Expected outcomes include changes in enzymatic activity (either increased or decreased), improved stability (three-dimensional structure of the enzyme), selectivity, and process control. Many factors can influence the catalytic function of immobilized lipases, which must exhibit selectivity to impart the desired specificity to the target product and provide maximum yield.^7,8^

Protein conformational stability can be significantly altered by immobilization, especially through interactions with silica surfaces. These changes may impact enzyme efficiency, either positively or negatively.^3,9^ A detailed understanding of the silicon chemical environments in silica-based materials is essential and can be achieved through ^29^Si NMR spectroscopy.^9,10^ In this way, the relative proportions of these sites can be quantified, and the influence of composition and environmental factors on the silica structure can be revealed, which can be described using Q sites, where Q*^n^* stands for a silicon atom bonded to *n* bridging oxygen atoms. The Q-sites, specifically Q^2^, Q^3^, and Q^4^, play a crucial role in determining the properties and behavior of silica-based materials.^6,9,11^

An effective and cost-efficient alternative to conventional methods of understanding the silica network, the different chemical shifts, and the structural information provided by the immobilization process is the combination of experimental and computational analyses.^12,13^ Therefore, knowledge of the interaction between lipase and immobilization carriers on silica-based supports is essential for effective application and represents a crucial area of research due to the potential applications of such immobilized enzymes in various industrial processes, including biodiesel production and ester synthesis.^8,14^

Future strategies for developing new generations of immobilized enzymes should leverage advancements in organic chemistry, and reactor design, emphasizing on computational chemistry and bioinformatics.^15^ Computational studies are promising in understanding support–enzyme interactions and quantifying the interactions to identify constraints and opportunities. These studies reduce analysis time and consumption of enzymes and reagents.^16^ Molecular docking is a tool for calculating the binding affinity of small molecules (ligands) and enzymes (receptors), which provides the molecular interactions of biocatalytic processes.^16,17^

Molecular docking can provide insight into enzyme selectivity, supporting the rational design of biocatalysts with enhanced performance for industrial applications. Future efforts must focus on engineering enzymes with increased selective promiscuity for diverse biotransformations, optimizing their performance to improve the cost-effectiveness and efficiency of industrial processes.^15,18^ These advantages motivated several studies on enzyme substrates to describe the behavior of small molecules that bind to target enzymes and clarify the molecular interaction mechanisms.^19,20^

This study used molecular docking to investigate the limitations of physically adsorbing *Burkholderia cepacia* lipase (BCL) onto a silica (SiO_2_) support. The computational predictions were validated through FTIR analysis and catalytic activity assays. To this end, docking simulations were carried out to elucidate how silica functional groups (Q^2^, Q^3^, and Q^4^) interact with specific residues in BCL.

Although progress has been made, the precise mechanisms by which silica functional groups interact with catalytic residues are still not fully understood. This work integrates experimental characterization and computational modeling to shed light on how enzyme–support interactions influence catalytic performance. These findings contribute to the rational design of more efficient immobilized enzyme systems.

The docking analysis identified key binding sites involved in the immobilization process. A multitechnique approach was used to investigate enzyme–support interactions at the molecular level, with special attention to catalytic site accessibility. This factor directly influences the performance of the immobilized biocatalyst in the esterification of butyl esters with fatty acids derived from *licuri oil*.

## Materials and Methods

### Materials

Licuri oil was provided by COOPES, Capim Grosso countryside, Bahia, Brazil (11°23′15.5″ S. 40°00′28.6″ W). The biocatalyst used was *B. cepacia* lipase (BCL, code 534641), purchased from Sigma-Aldrich (St. Louis, MO), and used without further purification. *N*-butanol and molecular sieve type 3 Å (form ball and size (0.3 nm)) was purchased from Vetec Química, Sigma-Aldrich, Brazil. All chemical reagents were of analytical grade.

### Enzymatic Hydrolysis of Licuri Oil

Free fatty acids from Licuri oil were obtained by enzymatic hydrolysis catalyzed by BCL following the procedure described by Rodrigues et al.^21^ The fatty acids were purified by successive washing with water and dried using anhydrous Na_2_SO_4_.^22^

### Preparation for Support

Silica matrix (SiO_2_) was prepared as described by Soares et al.,^23^ with some modifications. The sol–gel synthesis was performed using TEOS as precursor under an inert nitrogen atmosphere, and the polycondensation step was conducted at 4 °C for 24 h. The resulting material was then filtered, washed with *n*-hexane, dried in a desiccator, and sieved (32–60 mesh) prior to use.

### Enzyme Immobilization

The biocatalyst was immobilized on SiO_2_, adapting the methodologies described by Barbosa et al.,^24^ with minor modifications. The silica support was pretreated with *n*-hexane and subsequently incubated with the enzyme solution at room temperature, followed by 24 h at 4 °C to ensure adsorption. The hexane–aqueous interface was employed to facilitate interfacial activation of BCL.^25^ After immobilization, the biocatalyst was washed with *n*-hexane, dried in a desiccator, and stored at 4 °C until use. The immobilized protein loading was determined by the Bradford^26^ method and calculated according to Barbosa et al.^22^

### Characterization of Sol–Gel Support and Immobilized Biocatalyst

#### Structural Analysis by NMR Spectroscopy

The solid-state ^29^Si CP-MAS NMR spectra were obtained to evaluate the structural features of the silica support and the immobilized biocatalyst, following the procedure described by Soares et al.^9^ Spectra were recorded under standard CP-MAS conditions, and chemical shifts were referenced to Si­(CH_3_)_4_ and hexamethylbenzene.

#### Fourier Transform Infrared Spectroscopy (FTIR) and Deconvolution of FITR Spectra

FTIR analyses were performed using a Cary 630 FTIR spectrometer (Agilent Technologies) equipped with an ATR accessory. Subsequently, FTIR scans were recorded at room temperature (25 °C) to acquire the spectra of both the supports and the immobilized biocatalysts (Figure S1). Spectra were recorded over the range of 4000–500 cm^–1^, with 32 scans at a resolution of 4 cm^–1^. For secondary structure determination, the amide I region (1700–1600 cm^–1^) was selected due to its sensitivity to the C=O stretching vibrations of the protein backbone.^27^

Spectral processing included baseline correction, second-derivative analysis, and peak deconvolution. Four major peaks were identified and assigned to specific secondary structural elements: β-sheet (1610–1640 cm^–1^), random structure (1640–1650 cm^–1^), α-helix (1650–1658 cm^–1^), and β-turn (1660–1700 cm^–1^).^22^ Deconvolution was carried out using Gaussian curve fitting, and the integrated area under each peak was used to estimate the relative abundance of each structure.

Given the overlap between vibrational modes in the amide I region, FTIR does not enable the individual resolution of ordered/disordered helices or parallel/antiparallel β-sheets, as previously reported.^28,29^ Accordingly, these components were grouped into broader structural categories—α-helix, β-sheet, β-turn, and random structures—for quantification. Secondary structure contents are expressed as percentages relative to the total amide I absorbance.^30,31^

#### Thermogravimetric and Calorimetric Analysis (TGA/DSC)

Thermal stability of the silica support, free lipase, and immobilized biocatalyst was evaluated by TGA and DSC. Analyses were performed under a nitrogen atmosphere, following protocols previously described by Souza et al.,^32^ with heating up to 1000 °C. TGA curves were used to determine mass loss profiles, while DSC was applied to identify thermal transitions and calculate enthalpy values. The comparison among samples allowed the assessment of the effect of immobilization on enzyme stability.

#### Scanning Electron Microscopy and Energy Dispersive X-ray Spectroscopy (SEM/EDS)

Morphological and surface analyses of the samples were carried out using scanning electron microscopy (SEM) and energy dispersive X-ray spectroscopy (EDS). SEM imaging was performed using a field emission gun scanning electron microscope (FEG-SEM), JEOL JSM-7001F, operated to obtain high-resolution images of the sample surface. To minimize charging effects and improve image quality, the samples were coated with a thin layer of carbon alloy using a sputter coater prior to SEM analysis. Elemental analysis was conducted via EDS using an EDS-EDAX system with a Bruker AXS detector, managed by Quantax 200 software, Esprit version 1.9. To avoid signal interference from the conductive coating, EDS measurements were performed on uncoated samples, enabling accurate qualitative and semiquantitative analysis of the elemental composition and distribution. Linear scan modes were used to assess local variations across the sample surfaces. Linear scan modes were used to assess local variations in the surface elemental composition and morphology of the samples.

#### Textural Analysis (BET and BJH Methods)

The textural properties of the silica support and the immobilized biocatalyst were determined by nitrogen adsorption–desorption at 77 K. The Brunauer–Emmett–Teller (BET) model was applied to calculate specific surface area, while pore volume and pore size distribution were estimated using the Barrett–Joyner–Halenda (BJH) method, following IUPAC^33^ recommendations. Prior to analysis, samples were thermally treated at 120 °C to remove residual moisture. These measurements allowed comparison of surface accessibility and pore structure before and after enzyme immobilization.

#### X-ray Diffraction (XRD)

The XRD analysis was performed to investigate the structural features of the silica support before and after enzyme immobilization, following the procedure described by Oliveira et al.,^34^ using Cu–Kα radiation with modifications. The diffractograms were evaluated to confirm the amorphous character of the silica matrix and to identify possible structural modifications associated with lipase adsorption.

#### Molecular Docking Analysis

The structure of BCL (PDB ID: 3LIP) was obtained from Protein Data Bank (PDB) and the structures of the sites (Q^2^ = [Si­(OSi)_2_(OH)_2_], Q^3^ = [Si­(OSi)_3_(OH)] e Q^4^ = [Si­(OSi)_4_]) present in the silica was built using Discovery Studio 3.5 (Accelrys Software Inc., San Diego), following the composition described by Soares et al. Molecular docking simulations were carried out using AutoDock Vina 1.1.2 (The Scripps Research Institute, La Jolla, CA). The silica functional groups (Q2, Q3, and Q4) were treated as ligands, and BCL was used as the receptor. A grid box with dimensions of 108 × 118 × 122 Å was defined to fully encompass the enzyme surface, allowing for unrestricted identification of possible interaction regions across the protein. Each docking experiment was repeated ten times with different starting poses, and the conformation with the lowest binding energy was selected for analysis. AutoDockTools (ADT) was used to generate the rigid root of ligand (sites) and adjust the possible rotatable bonds (torsions), and well as to prepare the receptor structures.

#### Enzymatic Esterification and Butyl Ester Separation

Esterification activities and butyl ester separation were performed according to the methodology of Rodrigues et al.^35^ with modifications. Briefly, solvent-free reactions were performed with fatty acids from licuri oil and butanol at an equimolar ratio, using 10% (w/w) molecular sieve and 10% (w/w) biocatalyst at 45 °C and 200 rpm (Patent BR 10 2022 001442 6). The acid conversion (%) and enzymatic productivity (μmol·h^–1^·mg_protein_^–1^) were determined following established protocols by Miranda et al.,^36^ Lage et al.,^37^ and Alves et al.^38^ All the experiments were carried out in triplicate, and results are expressed as mean ± standard deviation. Negative controls (reactions without enzyme) were also performed to confirm the absence of nonenzymatic esterification. To ensure reproducibility, all reaction conditions were maintained constant, including stirring speed, temperature, and molar ratio.

#### Statistical Analysis

All experimental data are expressed as mean ± standard deviation from triplicate experiments (*n* = 3). Statistical comparisons between free BCL and immobilized SiO_2_–BCL were performed using Student’s *t* test for independent samples. The differences in conversion and productivity were statistically significant, confirming a substantial reduction in catalytic performance due to immobilization.

## Results and Discussion

This study combined complementary techniques better to understand the microenvironment of the enzyme–support interaction. NMR, molecular docking, and FTIR were applied jointly to investigate the enzyme–support interaction environment, correlating structural data with the catalytic performance of immobilized lipase in esterification reactions. BCL was immobilized via physical adsorption onto a silica (SiO_2_) support synthesized using the sol–gel technique, resulting in the biocatalyst referred to as SiO_2_–BCL. This immobilization protocol minimizes protein aggregation and promotes efficient dispersion of lipase molecules, enhancing their catalytic availability.^37,39^ The immobilized biocatalysts were prepared with an initial protein loading of 20 mg·g^–1^ of support. The maximum immobilized protein content was 17.72 ± 0.25 mg·g^–1^, corresponding to an immobilization yield of 88.60 ± 1.23%.

### Comparative Analysis of Immobilization Efficiency on Silica-Based Supports

To investigate the synergistic effect between the unmodified silica support (SiO_2_) and the lipase used in this study, a comparative analysis based on recent literature reports on the immobilization of lipase on silica-based materials was performed to evaluate the performance of our system in terms of immobilization efficiency and protein loading.

This comparative context is detailed below through a review of key studies involving the immobilization of lipases, especially BCL and *Pseudomonas cepacia* lipase (PCL), on a variety of silica-based supports. The discussion highlights how surface modifications, immobilization protocols, and material properties affect enzyme performance.

Immobilization of lipases on silica-based supports is a well-established strategy for enhancing biocatalyst stability, reusability, and catalytic efficiency in industrial applications such as biodiesel production and ester synthesis. Immobilization efficiency strongly depends on the type of silica support, surface modification, and immobilization technique employed. Studies have reported immobilization efficiencies above 70%, often reaching nearly 100% under optimized conditions.^40,41^

Mesoporous and hydrophobic supports tend to result in higher immobilization efficiencies, particularly when functionalized or addictive with hydrophobic groups such as octyl chains^42^ or combined with protic ionic liquids (PILs),^43^ which can prevent enzyme leaching and preserve structural integrity. PIL-modified silica aerogels have shown excellent performance for BCL immobilization. Lisboa et al.^44^ demonstrated that BCL immobilized on silica aerogels with protic ionic liquids exhibited improved activity recovery, better enzyme dispersion, and greater structural stability compared to the unmodified support. Adsorption, covalent bonding, and cross-linking methods also influence the final performance of the immobilized enzyme.^45,46^ Furthermore, parameters such as pH, protein concentration, and support porosity play essential roles in maximizing immobilization outcomes.^41^

While many studies emphasize the advantages of support modification for enzyme immobilization, recent findings by Lisboa et al.^57^ reported that unmodified silica aerogel, used as a control for BCL immobilization, exhibited only 66.8% immobilization efficiency and 63.5% activity recovery. In contrast, our unmodified mesoporous silica achieved 88.6% immobilization efficiency and a protein loading of 17.72 mg·g^–1^, while maintaining comparable catalytic activity. These results demonstrate that high performance can also be achieved with simpler, unmodified systems. This reinforces the potential of unmodified mesoporous silica as a cost-effective and robust support for biocatalyst development.

Although surface modification with specific functional groups is often recommended to improve the performance of immobilized lipases, hybrid supports incorporating lignocellulosic biomass as organic components may offer a sustainable and effective alternative.^47^ Nevertheless, future work may benefit from support hybridization strategies to further enhance enzyme conformation, accessibility, and stability.

### Analysis of the Structure of SiO_2_–Pure and SiO_2_–BCL by NMR

According to Soares et al.,^9^ the Si–OH functional groups detectable by NMR^29^Si can interact with binding sites of BCL. The NMR^29^Si spectra were interpreted based on the characteristic chemical shifts of Qn sites. Q^2^, Q^3^, and Q^4^ exhibit distinct chemical shifts, enabling their identification. In general, Q^2^, Q^3^, and Q^4^ signals are observed within the −80 to −120 ppm range.^11,48^ Figure 1 shows the spectra of pure NMR^29^Si and NMR^29^Si–SiO_2_–BCL, with characteristic peaks between 80 and 120 ppm. After lipase adsorption, the reduction of Q^2^ and Q^3^ sites, accompanied by an increase in Q^4^ content, suggests greater condensation of the silica network with fewer accessible silanol groups. The **s**imilar assignments and interpretations of the Q*^n^* distribution in silica networks the findings of He et al.,^49^ who related higher Q^4^ content to more condensed and thermally stable silica structures. The SiO_2_ units are derived from the hydrolysis of TEOS, and in the case of the pure silica matrix, the relative proportions of Q^2^, Q^3^, and Q^4^ sites were 5.03%, 58.66%, and 36.21%, respectively (Figure 1A).

**Figure 1:**
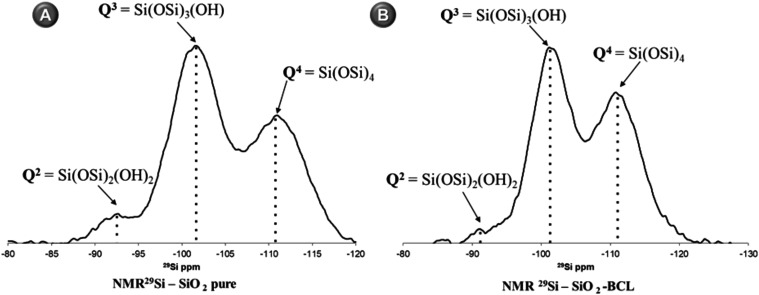
(A) NMR^29^Si spectra of the SiO _2_ (pure silica) and (B) SiO_2_–BCL (BCL adsorbed), which exhibit distinct chemical shifts for the peaks between 80 and 120 ppm.

The functional groups Q^2^ (0.96%) and Q^3^ (44.43%) of NMR^29^ Si–SiO_2_–BCL were smaller than those of pure silica matrices, which were less than 50%, as shown in Figure 1B. The observed decrease in the relative percentage sum of Q^2^ and Q^3^ sites indicates a reduction in the availability of Si–OH groups in the matrices, which can be correlated with enzymatic activity. These data are consistent with each other, as a more densified silica network with a higher Q^4^ peak intensity is expected to have a smaller specific surface area for matrices derived from TEOS. The NMR^29^Si data and the specific surface area data demonstrate the presence of interactions between the enzymes and the silica network.

According to Matuella et al.,^4^ the surface of the TEOS gel support (pure silica) and the lipase entrapped in the gel exhibited extremely low porosity or a virtually nonporous structure. Moreover, Buisson et al.^50^ *B. cepacia* lipase accelerates gelation kinetics and modifies the gel structure, producing more silicon sites in silica.

### Physicochemical and Morphological Characterization of Biocatalysts

#### Morphological and Elemental Characterization by SEM and EDS

The morphological characterization by scanning electron microscopy (SEM) revealed distinct differences between pure SiO_2_,the immobilized enzyme system (SiO_2_–BCL) (Figure 2A, B and D, E), and Energy-dispersive spectroscopy (Figure 2C, F). Pure SiO_2_ exhibited a homogeneous surface with well-defined, open pores, a typical feature of sol–gel-derived materials, which ensures a high surface area suitable for enzyme binding (Figure 2A, B).^51,52^ In contrast, SiO_2_–BCL composite displayed an irregular surface morphology (Figure 2D, E), with partial pore coverage and visible enzyme aggregates, thereby confirming the successful immobilization of lipase onto the silica matrix.

**Figure 2:**
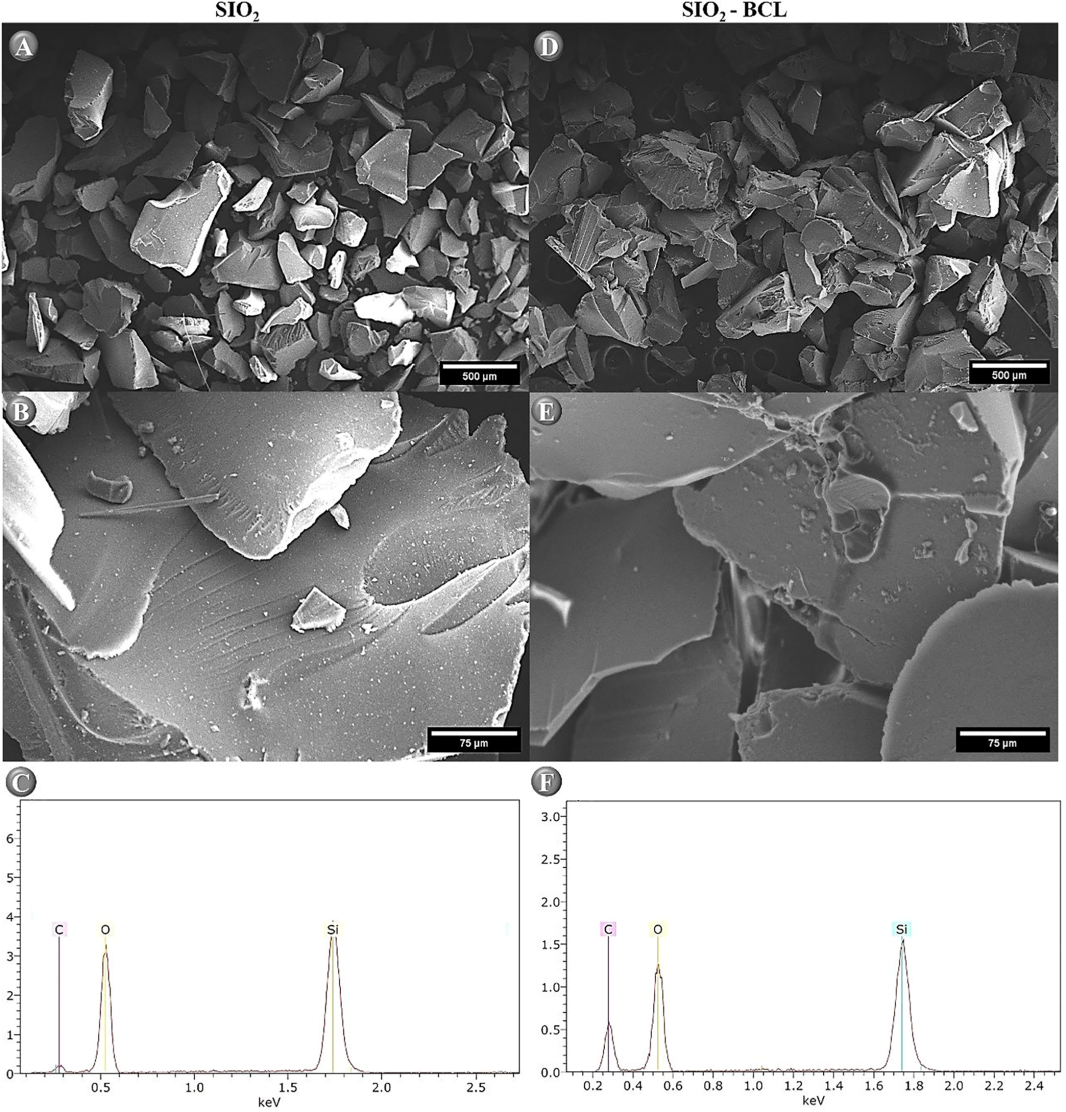
Scaning electron micrographs for: (A) SiO_2_ (×300), (B) SiO_2_ (×500), (D) SiO_2_–BCL (×300), and (E) SiO_2_–BCL (×500). Energy-dispersive spectroscopy for: (C) SiO_2_, and (F) SiO_2_–BCL.

Carvalho et al.^43^ analyzed the SEM micrographs of BCL immobilized on a silica xerogel support and modified with an ionic liquid and found morphological differences between pure SiO_2_ and immobilized lipase, such as low surface porosity, due to the absence of a functional agent.

Complementary to the SEM analysis, energy-dispersive spectroscopy (EDS) provided insights into the elemental composition changes induced by the immobilization process. The carbon signal increased from 11.07% in pure SiO_2_ to 33.37% in SiO_2_–BCL, reflecting the contribution of the protein to the composite. The predominant elemental signals of silicon and oxygen remained, indicating that the silica framework was structurally preserved. In the EDS analysis, the morphological structures of the support and the biocatalyst are described by Girelli et al.,^47^ showing a significant increase in oxygen (O) content by about 20% after enzyme adsorption, as also observed for SiO_2_ and SiO_2_–BCL in Figure 2C,F. Consequently, the carbon (C) content, which is not initially present in the support, also increases and can be attributed to the presence of enzyme aggregates adsorbed onto the silica surface.

However, the proportion of silicon decreased from 30.67% to 18.61%, consistent with partial surface coverage by the enzyme. These combined morphological and elemental findings confirm the effective lipase immobilization on the silica xerogel, with potential implications for catalytic performance and stability.

#### Structural Analysis by X-ray Diffraction (XRD), Specific Surface Area and Porous Properties

X-ray diffraction (XRD) analysis was performed to evaluate possible structural changes in the silica matrix (SiO_2_) after immobilization of *B. cepacia* lipase (SiO_2_–BCL). The XRD patterns of SiO_2_ and SiO_2_–BCL (Figure 3A) show a characteristic broad band centered around 22°-25° (2θ), typical of amorphous materials produced by the sol–gel process.^53,54^ This broad signal reflects the absence of long-range order in the Si–O–Si network, a feature generally associated with xerogels obtained by this method.^55^

**Figure 3:**
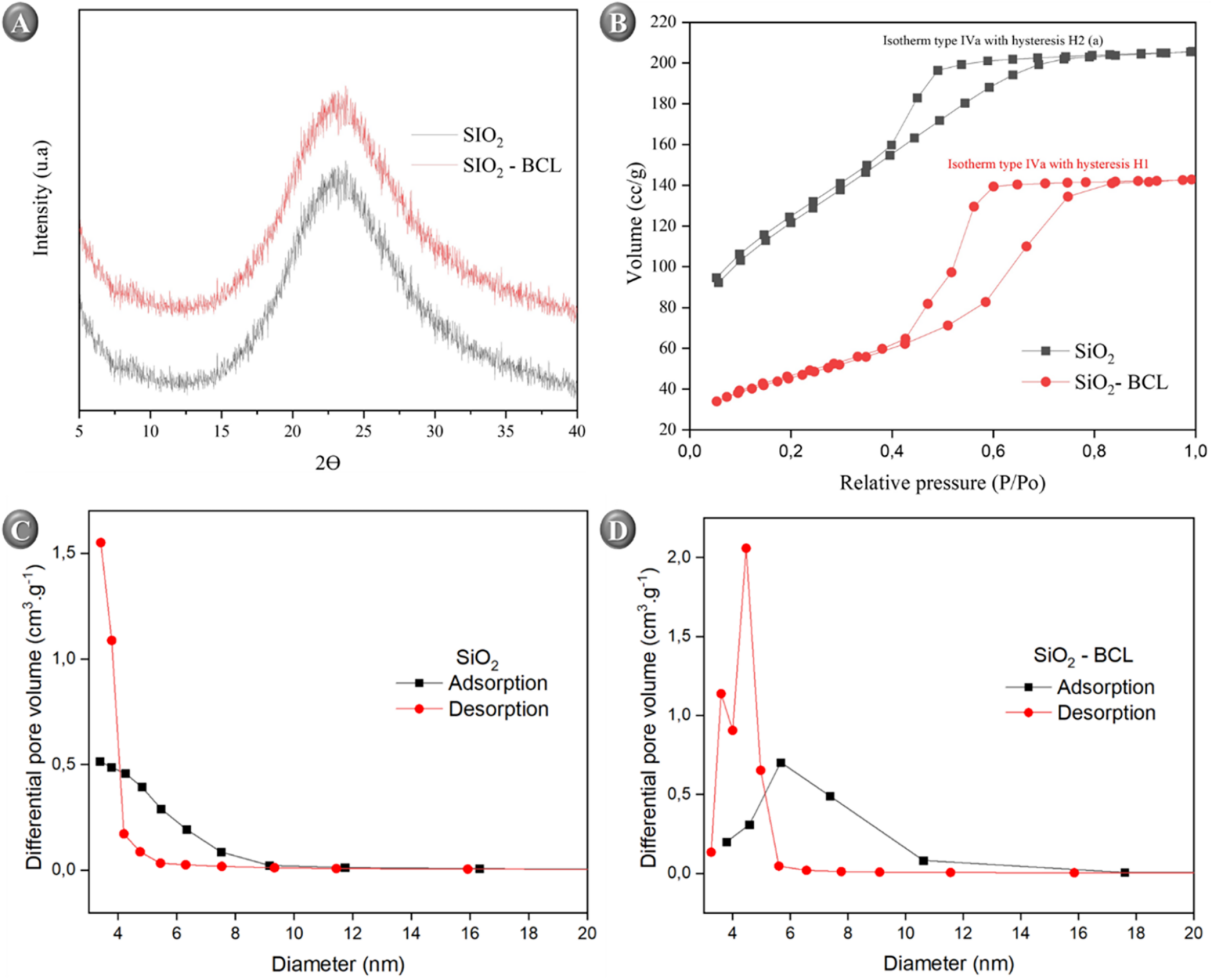
(A) X-ray diffractograms of SiO_2_ and SiO_2_–BCL, (B) Nitrogen adsorption–desorption isotherms of the pure silica gel and immobilized sample. Pore size distribution for: (C) SiO_2_, (D) SiO_2_–BCL.

After enzymatic immobilization, it was observed that the amorphous pattern was maintained without evidence of new crystalline reflections or structural degradation. Similar to Deon et al.,^56^ XRD analysis revealed that immobilization of the lipase on the silica matrix did not lead to any significant structural changes in the support, and the characteristic amorphous pattern with a broad diffuse band was maintained. As expected, no additional peaks attributable to enzyme crystallization were detected, since proteins typically do not produce sharp diffraction signals. Owoeye et al.^57^ also reported a single broadband peak between 15° and 30° was identified for that material.

The characterization of porosity in hydrophobic matrices and immobilized biocatalysts is a complex challenge requiring detailed analysis of total porosity, pore size, and pore size distribution. Gas adsorption-based methods stand out for their convenience in evaluating the porous properties of solid materials using volumetric measurements of adsorbed gas quantities. In this study, nitrogen (N_2_) adsorption–desorption isotherms at −196 °C (Figure 3B) were employed to determine the specific surface area (SSA), specific pore volume (*V*_p_), and average pore diameter (*d*_p_) of the hydrophobic matrices and immobilized biocatalysts, with values presented in Table 1.

**Table 1:** Textural Properties from Nitrogen Adsorption–Desorption of the Pure Silica Gel (SiO_2_) and Immobilized Sample (SiO_2_–BCL)

samples	surface area (m^2^·g^–1^)	*pore volume (cm^3^·g^–1^)	pore diameter (nm)
SiO_2_	431.642	0.148	3.8
SiO_2_–BCL	163.399	0.193	4.4

The BET method was used to determine the surface area, while pore size distribution was evaluated by the BJH method, commonly applied for the analysis of micro- and mesoporous solids.^49^ Results show that BCL caused marked structural alterations in the silica matrices. The specific surface area of the support matrix (SiO_2_) drastically decreased from 431.6 to 163.4 m^2^/g, a reduction of approximately 62%. This reduction is attributed to partial pore filling or blockage by the enzyme, restricting adsorbate access and representing a typical effect of sol–gel-based immobilized systems.^58^

Conversely, a slight increase in total pore volume from 0.148 to 0.193 cm^3^/g, which may reflect structural rearrangements or the relative expansion of larger pores upon enzyme adsorption. Comparative analysis of the isotherms revealed a transition in hysteresis type from H2 to H1, suggesting a transition toward more uniform cylindrical pores with narrower distribution, conditions that can improve diffusion and substrate accessibility to the active sites.^33^

As reported by Pavan et al.,^59^ although SEM does not show significant differences in aggregated particle size at different organic precursor concentrations, increasing organic content reduces in surface area due to pore coverage and preferential blockage of larger pores. Pore size distribution analysis confirms this effect, showing a reduction in the proportion of larger pores and a shift toward smaller pore sizes (∼3.5 nm), thereby modifying matrix topology and accessibility.

The pore volume and surface area distribution determined by the BJH method (Figure 3C, D) showed apparent structural differences between samples. In the SiO_2_ support, the pore volume is dominated by smaller pores (∼1.7–2.7 nm) with a correspondingly large surface area. In comparison, in the SiO_2_–BCL sample, these smaller pores exhibit lower volume and surface area, reflecting the adsorbed enzyme’s partial filling and clogging of the channels.

Moreover, the adsorption–desorption isotherm analysis (Figure 3B) exhibits classic hysteresis behavior, with higher volume and surface area during desorption compared to adsorption, characteristic of mesoporous pores with “ink-bottle” geometry and tortuous channels (H2-type hysteresis for SiO_2_). After immobilization, a transition to H1-type hysteresis is observed, indicating more regular cylindrical pores with a narrower size distribution favorable for molecular diffusion and substrate access to enzyme.^56,60^

The surface area and pore volume values obtained here for the silica xerogel are consistent with those reported by Maseko et al.,^61^ who described surface areas near 668 m^2^·g^–1^ and pore volumes around 1.26 cm^3^·g^–1^ for similar materials. Percentage differences can be attributed to variations in synthesis methods and experimental conditions but remain within the typical range for sol–gel xerogels.

Additionally, Linsha et al.^60^ highlight that although xerogels exhibit an unimodal distribution dominated by small pores and an ink-bottle structure, this limitation is compensated by greater structural stability and compatibility for enzyme immobilization.

Changes in porous properties and surface area following enzyme immobilization directly affect lipase activity and stability. The reduction in surface area indicates fewer free binding sites; however, the relative opening of larger pores and more regular pore geometry facilitate substrate and product transport, optimizing catalytic efficiency.^10,62^ Furthermore, the hydrophobic environment of the SiO_2_–BCL support helps maintain the enzyme’s active conformation, contributing to enhanced performance.

#### Thermal Analysis of Materials by DSC and TGA

The weight loss of pure silica (SiO_2_), biocatalyst-free (BCL), and immobilized (SiO_2_–BCL) samples was determined by thermogravimetric analysis (TGA). The weight loss obtained after heating the samples to 1000 °C is shown in Table 2. The results showed that the SiO_2_ sample exhibited a weight loss of only 12.34%. This loss can be attributed to unreacted silanol groups from TEOS, resulting from incomplete sol–gel reactions, characteristic of predominantly inorganic materials.

**Table 2:** Total Loss of Mass, and Enthalpy of the Pure Silica, Free Enzyme and Immobilized of Lipase from *B. cepacia*

		weight loss (%)				
	regions	I	II	III	total weight loss (%)	AH (J·g^–1^)
samples	BCL	6.82	70.58	4.10	81.50	528,27
	SiO_2_	8.18	2.96	1.20	12.34	377.55
	SiO_2_–BCL	7.56	10.74	2.29	20.59	331.38

Additionally, part of this weight loss is due to the removal of tightly bound water molecules.^52^ The BCL sample exhibited a mass loss of 81.50%, whereas the SiO_2_–BCL sample showed an average loss of 20.59%, indicating the presence of the incorporated enzyme fraction. The TGA curves for all samples are presented in Figure 4A–C. The matrix with immobilized lipase exhibited approximately 8% higher weight loss than the other samples (Figure 4C), which may be attributed to the greater volume of BCL. As Soares et al.^9^ suggest, higher weight losses observed with immobilized matrices correlate with increased thermal stability, which results from interactions between silane precursors and organic components, such as lipase.

**Figure 4:**
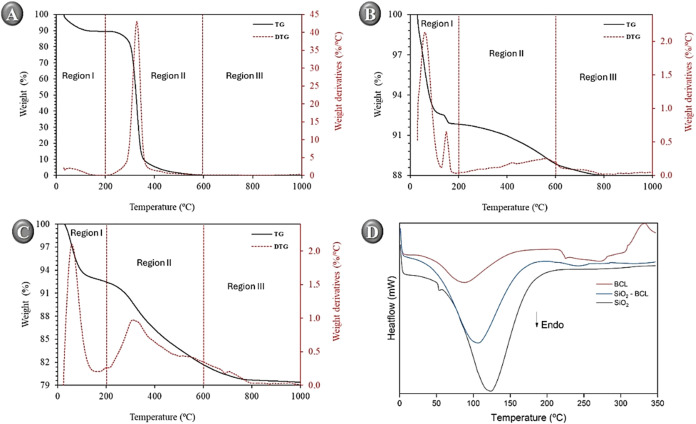
TG/DTG curve of samples (A) BCL, (B) SiO_2_ and (C) SiO_2_–BCL at 20 °C·min^–1^ under nitrogen atmosphere. DSC curves at 10 °C·min^–1^ under nitrogen atmosphere of all samples.

The thermographs were divided into three regions. Region I (below ∼200 °C) corresponds mainly to dehydration, involving the release of surface water and loosely bound groups within the sol–gel matrix. Region II (200–600 °C) is characterized by condensation of silanol groups and degradation of organic components (C, H, O, N), consistent with protein decomposition processes such as lipase denaturation. In region III, the weight loss is associated with the advanced hydroxylation and carbonization of residual organic matter, including the enzyme.^49^ Above ∼750 °C, the material reaches thermal stability, and complete decomposition occurs, as observed for the free biocatalyst sample (Figure 4A).

The reduction in mass loss in the immobilized sample, compared to the free enzyme, indicates a stabilizing effect provided by the silica matrix, which acts as a barrier to thermal degradation. This behavior is consistent with the findings reported by Zhou et al.,^63^ and Ashkan et al.,^64^ who emphasize immobilization as an effective strategy to enhance the thermal stability of enzymes, thereby reducing their susceptibility to thermal denaturation and protein oxidation at elevated temperatures.

Complementary to the TGA analysis, Differential Scanning Calorimetry (DSC) monitors heat flow associated with controlled heating of a sample. This technique allows the identification of phase transitions and thermal events, which appear as endothermic or exothermic peaks in the DSC profile.

The sample containing the lipase from *B. cepacia* (Figure 4D) showed an initial endothermic event at 86 °C (Δ*H* = 528.27 J·g^–1^), followed by minor signals attributed to water release and degradation of organic residues. The pure silica (SiO_2_) sample (Figure 4D) showed a single endothermic transition at 125 °C with Δ*H* = 377.55 J·g^–1^. Comparing samples with and without lipase, the DSC curves corroborate the TGA data, showing distinct endothermic profiles. Free BCL exhibited a strong endothermic peak corresponding to the transition, whereas the SiO_2_–BCL sample showed a weaker peak with an enthalpy of 331.38 J·g^–1^ and an even lower value. This reduction in enthalpy in the immobilized sample reflects the conformational restriction of the enzyme within the matrix, limiting the amplitude of its structural movements, such as loops and catalytic domains.

As described by D’amico et al.,^65^ the lower enthalpy observed in immobilized enzymes indicates reduced structural mobility and a conformational state requiring less energy for thermal denaturation. These findings align with the hypothesis that immobilization induces a “partial stiffening” of the protein structure, potentially affecting stability and catalytic activity.

In the present study, BCL exhibited an activity of 1500 U and a thermal transition enthalpy (Δ*H*) of 528.27 J·g^–1^, significantly higher than the values reported by Souza et al.^52^ for the same enzyme, which were 870 U and 177.7 J·g^–1^, respectively. This marked difference in enthalpy suggests greater conformational stability of the enzyme studied, which correlates directly with the enhanced catalytic activity observed. This is consistent with the report by Petrović et al.,^66^ who highlight the influence of cooperative motions far from the active site on enzymatic reactivity and note that enzymes sharing identical active site structures may exhibit differing catalytic efficiencies depending on their global conformational dynamics.

Therefore, the data obtained here confirms a strong correlation between catalytic activity and thermal stability. Thermodynamic analysis (Δ*H*), combined with mass loss data and thermal profiling, is a powerful approach to evaluate the quality and functionality of different enzyme batches, enabling indirect structural insights into the protein’s conformational state and the efficacy of the immobilization strategy employed.

#### Computational analysis of *B. cepacia* Lipase Immobilized in Silica

The ^29^Si NMR can quantify the relative proportions of Q^2^, Q^3^, and Q^4^ species in silica samples, providing a detailed understanding of the silica network structure. Corroborating the above, we employed molecular docking analyses to elucidate the interaction mechanism between silica functional groups (Q^2^, Q^3^, and Q^4^) and lipase binding sites (BCL). This approach allowed it to identify the specific lipase binding sites to which the silica functional groups could bind during immobilization. Notably A more negative binding energy value corresponds to a stronger interaction between the support and the lipase. Table 3 presents the data predicted by molecular docking analyses, showcasing the lowest absolute affinity value (kcal·mol^–1^) and the interactions between Si sites and amino acids residues in the active site of the lipases.

**Table 3:** Docking Affinity Energy and Individual Interactions of Functional Groups of Silica with the Active Site of the BCL Predicted by AutoDock Vina

sites	afinity (kcal·mol^–1^)	interactions with amino acids residues of the active site	types of interaction
Q^2^	–10	Asn48	hydrogen bond
		Asn59	
		Gln34	
		Asp21	
		Asn59	
		Tyr68	
		Tyr68	
Q^3^	–9,4	Thr18	hydrogen bond
		**Ser87**	
Q^4^	–8,5	Arg40	hydrogen bond
		Thr280	
		Thr280	
		Ser281	
		Tyr282	
		Thr310	
		Ser281	

The docking poses with the lowest absolute affinity values and specific interactions for each group anchored to the BCL are shown in Figure 5. Analyzing the interaction energies of the functional groups with the catalytic triad of the BCL, which consists of the amino acids residues serine87 (Ser87), aspartic acid264 (Asp264), and histidine286 (His286),^8^ the data showed that the Q^2^ and Q^4^ groups had interaction energies with BCL (−10 and −8.5 kcal·mol^–1^, respectively) after interacting with several amino acids residues through hydrogen bonds, which is the best conformation for the stability of the biocatalyst in catalytic activity. Even though Q2 and Q4 formed hydrogen bonds (Figure S2) with residues like Asn48 and Arg40, they did not interact with the catalytic triad (Ser87, Asp264, His286), which could explain why they had little to no impact on catalytic activity. These interactions happen in regions away from the active site or are positioned in a way that does not interfere with substrate access, unlike what is observed with the Q3–Ser87 interaction.

**Figure 5:**
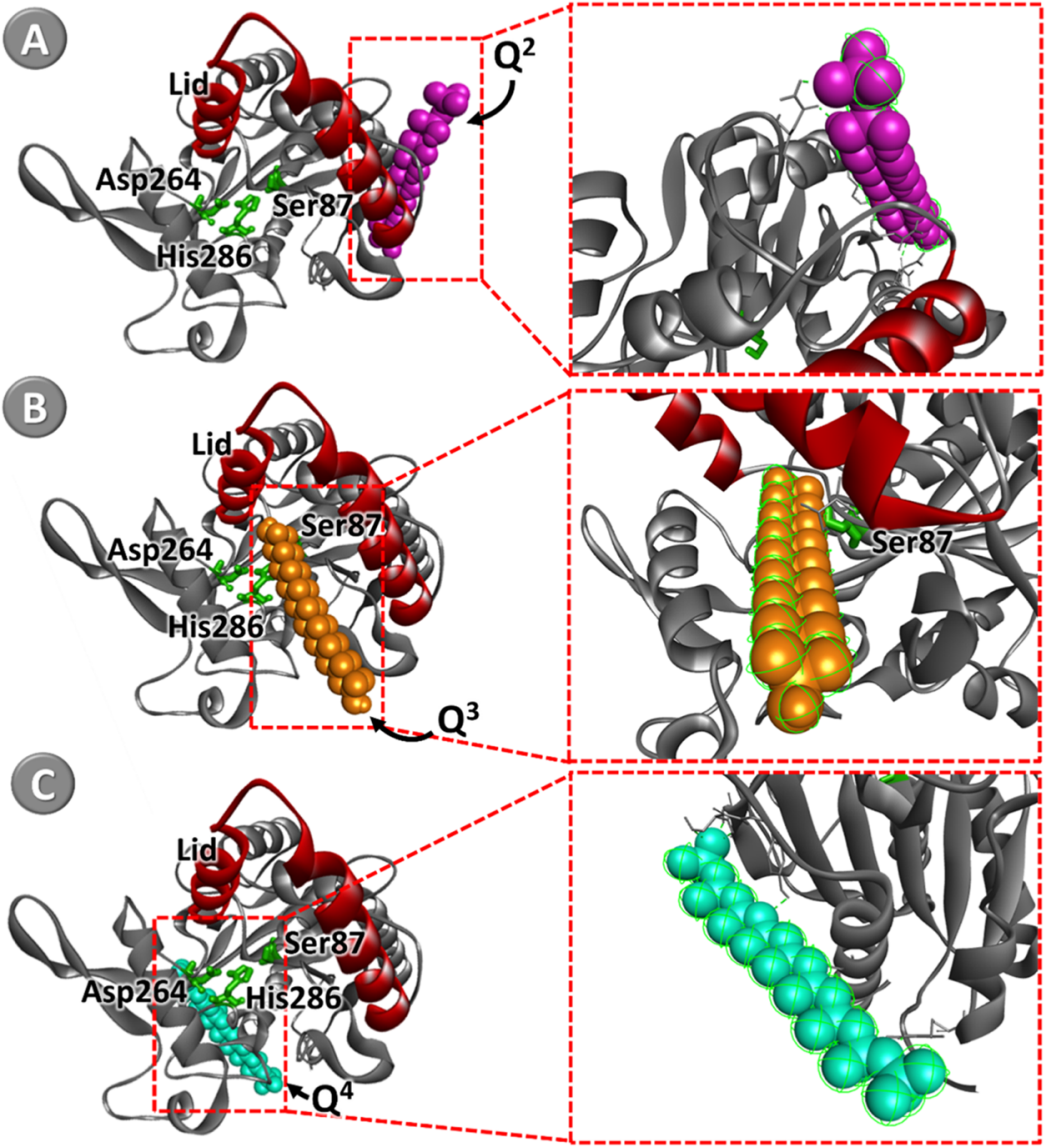
Molecular docking of the sites (A) Q^2^, (B) Q^3^, and (C) Q^4^ in the BCL structure (lipase catalytic triad in green (Ser87, Asp264, and His286)) and lid in red.

It was observed that only the Q^3^ group interacted with the amino acid of the catalytic triad (ser87), requiring an energy of −9.4 kcal.mol^–1^for the interaction, possibly limiting the access of the substrate to the catalytic triad (Figure 5B).

When analyzing the computational simulations, the interaction of the Q^3^ group in the active site of the BCL can be observed, which can hinder or facilitate the access of the substrate, affecting the enzymatic activity. It is possible to observe in docking poses (Figure 6A, D) that Q^3^ is located close to the secondary structures (α-helix and β-sheet) of the BCL (Figure 6C), which indicates the displacement of the lid upon conformational changes.^36,67^ The closer to the α-helix and β-sheet of the lid domain, the smaller the displacement and the less exposed the active site of the enzyme, making the BCL structure stiffer due to the loss of intermolecular hydrogen bonds between the water molecules and the surface of the enzyme.^29^

**Figure 6:**
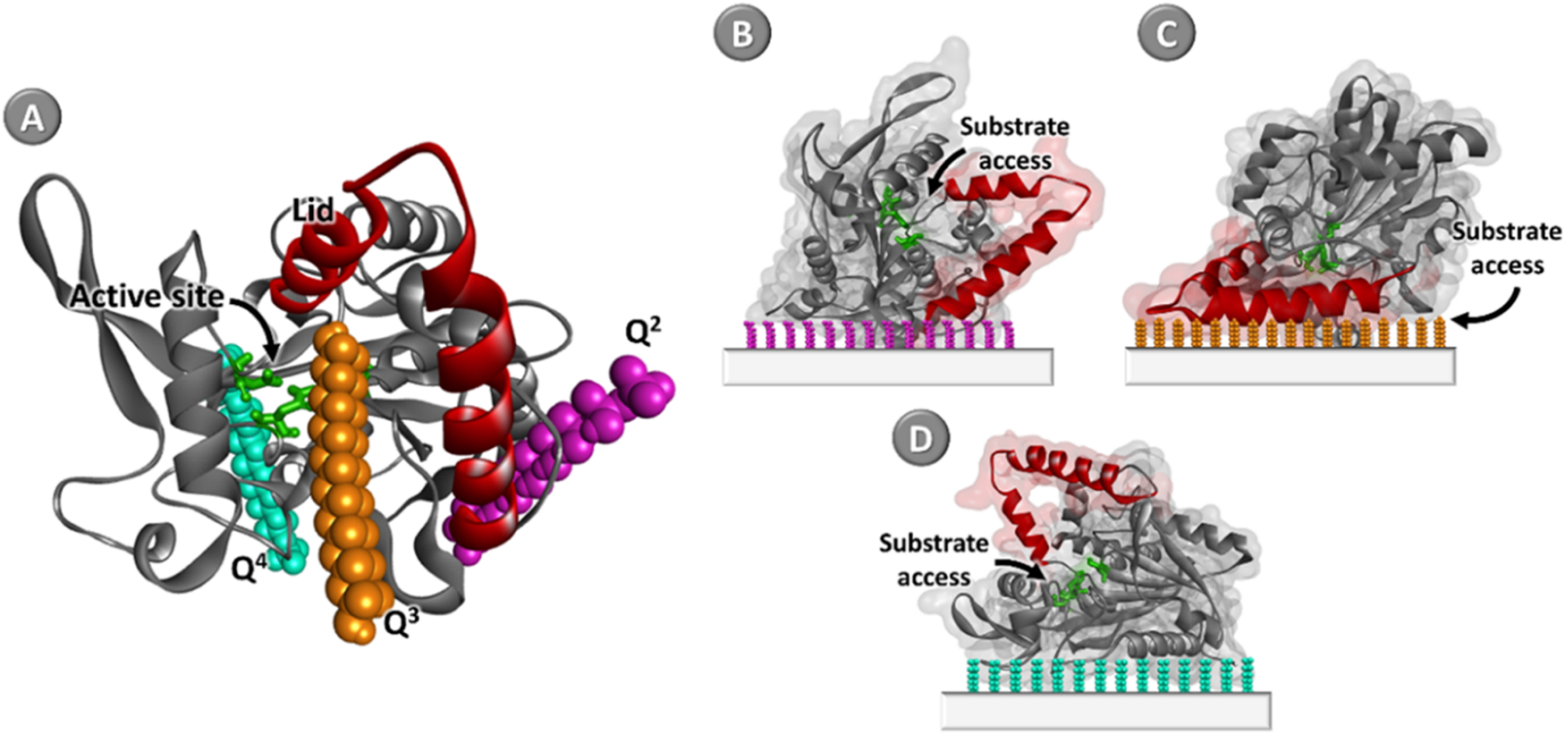
(A) Three-dimensional structure of *B. cepacia* lipase (BCL, PDB ID: 3LIP) immobilized on the silica surface, (B) Q^2^ (purple), (C) Q^3^ (orange), and (D) Q^4^ (blue) representing the spatial positioning of access active site of the biocatalyst immobilized.

Although molecular docking provides valuable insights into the interactions between enzymes and surfaces, it is important to point out that this technique is a simplified model that does not fully account for the dynamic nature of proteins and the effects of solvents. Nonetheless, the experimental data in this work confirmed the main predictions, underpinning the reliability of the computational analysis to describe binding affinity and active site accessibility.

#### Fourier Transform Infrared (FTIR) Spectrometric Secondary Structure Analysis

Due to the docking analysis suggested that Q^3^ is located near the secondary structures of BCL, the FTIR study was performed in the amide I region (1700–1600 cm^–1^) to confirm the structural changes of BCL after immobilization and to define its secondary structure as this region is sensitive to conformational changes. Thus, β-sheet (1610–1640 cm^–1^), random structure (1640–1650 cm^–1^), α-helix (1650–1658 cm^–1^), and β-turn (1660–1700 cm^–1^) were the components of this region analyzed in the secondary structure of SiO_2_–BCL, as shown in Figures 7A, B.

**Figure 7:**
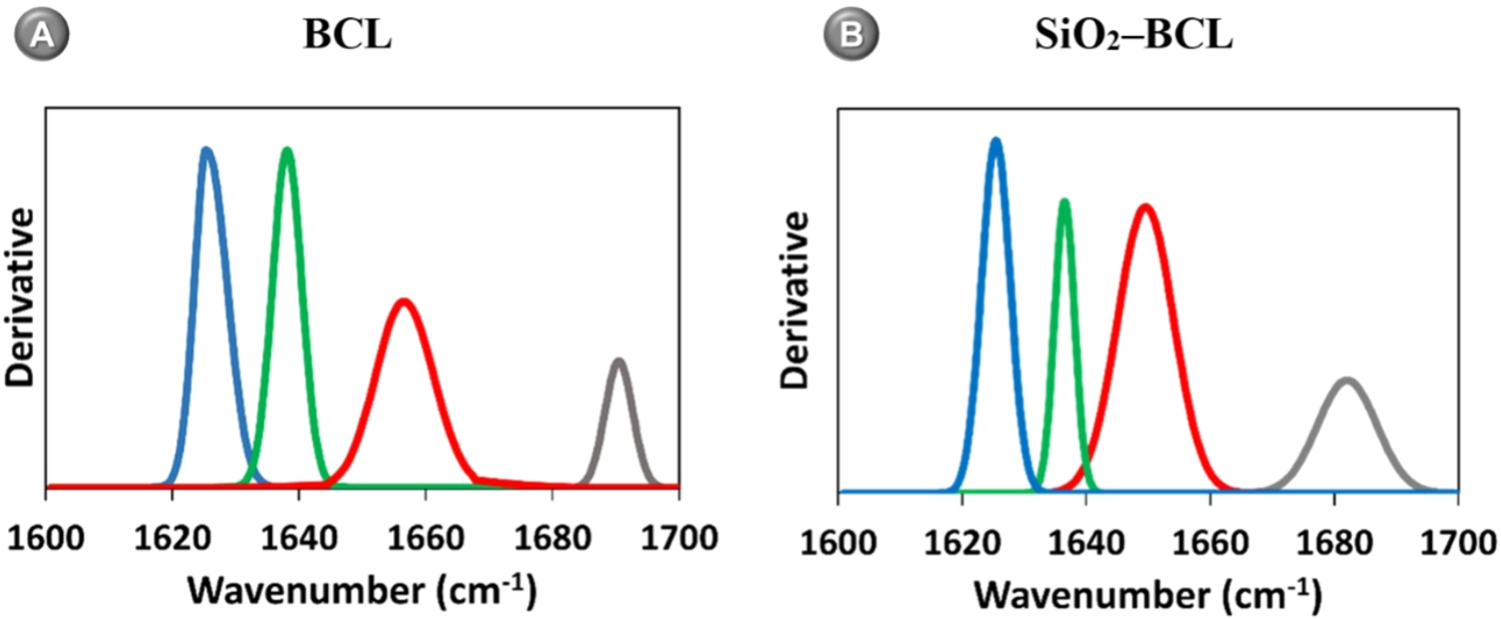
FTIR Spectra was derived from the amide I region (1700–1600 cm^–1^) to confirm the structural changes of (A) BCL and after immobilization and (B) SiO_2_–BCL. Thus, β-sheet (1610–1640 cm^–1^), random structure (1640–1650 cm^–1^), α-helix (1650–1658 cm^–1^), and β-turn (1660–1700 cm^–1^) were the components of this region analyzed.

The data shows that the synthesis process of the biocatalyst led to changes in the secondary structure of BCL. These changes are most evident when analyzing the α-helix content, as a significant increase in SiO_2_–BCL was observed.

Thus, the data of the area (%) on the secondary structure by FTIR shows that the percentage of secondary structure elements changed after the immobilization of BCL. In free BCL, the rate of α-helix was 28.46%. Comparing the spectra shown in Figure 7A before and (B) after immobilization, there is an increase in the content of SiO_2_–BCL α-helix (37.30%), and as presented in the docking, it is likely that the silica (SIO_2_) has a direct interaction with the structure of the lipase and partially hinders the access of the substrate to the active site of BCL.

This change plays an important role in the catalytic activity of BCL and correlates with the possibility of substrate access to the enzyme’s active site.^7^ Some authors have shown that an increase in the α-helix content makes it more difficult for the substrate to access the active site of the lipase.^29,30^

Changes in the secondary structures of enzymes, such as α-helix and β-sheet, play a critical role in their functionality and stability. Immobilization can alter the balance between these structures, often enhancing enzyme stability and catalytic efficiency by providing greater structural rigidity, which increases resistance to denaturation. Additionally, modifications in random coils and β-turns, contribute to the enzyme’s flexibility can influence substrate binding and catalytic performance. These structural adjustments upon immobilization may lead to improved substrate accessibility and higher turnover rates, further optimizing enzymatic activity.^68^

The knowledge of the secondary structure is helpful but needs to be more detailed to fully correlate changes in a specific activity to a change in the protein structure.^3^ Additionally, molecular docking simulations have shown that enzyme orientation is important for enzyme activity, as it affects the accessibility of substrates to the active site.

#### Catalytic Performance of Biocatalysts in the Esterification of Licuri Oil Free Fatty Acids

Molecular docking simulations and FTIR analyses were important for predicting the intermolecular interactions between the functional groups of silica and BCL and highlighting the changes in secondary structure conformation, respectively. Still, it should be remembered that the appropriate selection of a support matrix is directly related to the type of enzyme and the process in which it will be used, so experimental trials are essential to validate the predicted data.

Thus, according to the methodology described by Rodrigues et al.,^21^ the free fatty acids produced in the hydrolysis reaction were subjected to the purification process to eliminate any impurities. Subsequently, the FFA was applied in the esterification process with butyl alcohol to evaluate the catalytic performance of BCL and SiO_2_–BCL. The biocatalysts were used separately in stirred tank reactors containing *licuri oil* and distilled water (25% m/m mass ratio of oil to water) for 24 h.

Figure 8A, B presents the efficiency of BCL and SiO_2_–BCL in ester synthesis based on conversion and productivity. This data provides a comprehensive understanding of the performances of the biocatalysts applied in the reaction, aiding in the evaluation of their effectiveness and potential applications.

**Figure 8:**
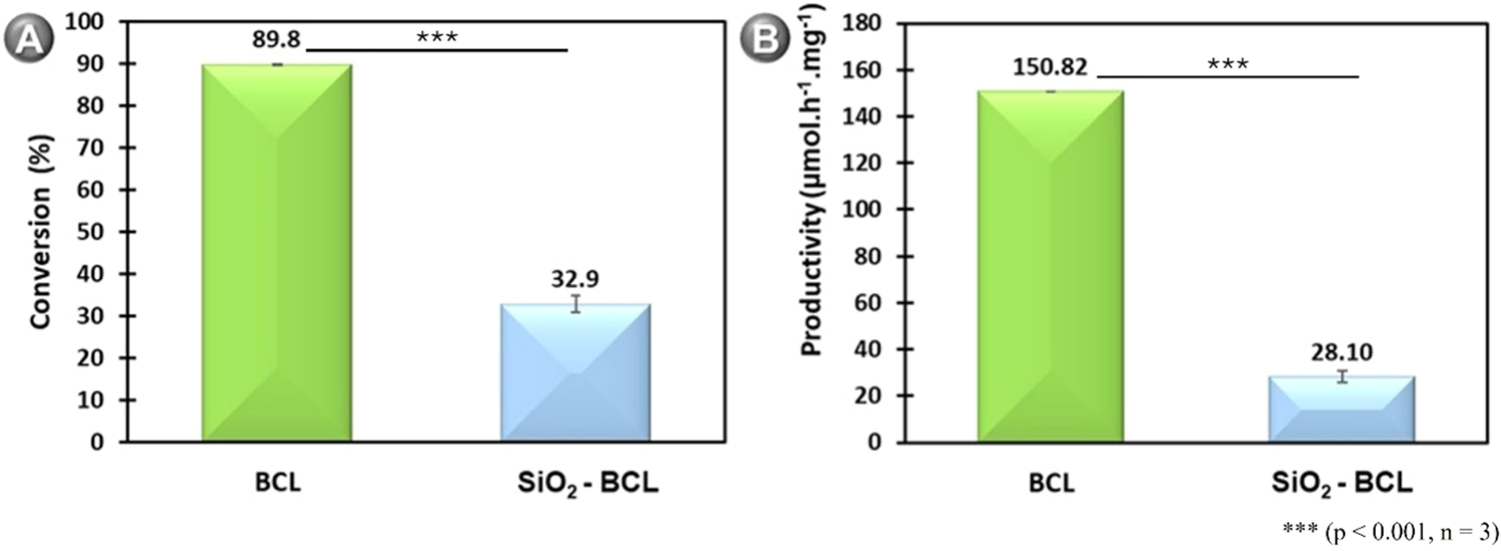
Efficiency of biocatalysts BCL, and SiO_2_ – BCL in ester synthesis on (A) conversion and (B) productivity. In a solvent-free system with fatty acids from Licuri oil, butyl alcohol, with an equimolar ratio, a molecular sieve (10% w/w) around 10 mg_protein_/g_enzyme_, and biocatalysts (10% w/w), at 45 °C and 200 rpm for 24 h. Bars represent mean ± standard deviation (*n* = 3). Statistical significance was assessed by Student’s *t* test. ***p < 0.001.

It was possible to note that the esterification activity (498.00 ± 30.62 μmol·h^–1^·mg^–1^) using the SiO_2_–BCL biocatalyst is almost three times lower compared to BCL (1360.39 ± 41.49 μmol·h^–1^·mg^–1^) in free form. These reductions in both conversion and productivity were statistically significant, as confirmed by Student’s *t* test (*p* < 0.001, *n* = 3). As expected, BCL and SiO_2_–BCL exhibited different catalytic efficiencies, as the molecular docking simulations revealed that the Q_3_ functional group of SiO_2_ preferentially docks to the amino acid ser87, which forms the catalytic triad of the enzyme, that is, it likely causes a drop in performance of the immobilized biocatalyst and limits its catalytic performance during ester synthesis.

Thus, the molecular docking data correlate positively with the experimental tests and show that this preference may hinder access to the substrate and affect enzyme activity, as SiO_2_–BCL did not achieve good efficiency in the esterification reaction. Although previous studies have shown that increasing the hydrophobicity of the support can increase lipase activity by improving substrate access,^69^ in the present work, despite its hydrophobic nature, the Q^3^ group interacts directly with the catalytic residue Ser87, which appears to hinder access to the active site and reduce enzymatic performance.

As shown in Figure 8A, the BCL, in its free form, significantly outperformed SiO_2_–BCL, which shows the maximum degrees of esterification using BCL (89.76 ± 0.71%) and SiO_2_–BCL (32.86 ± 0.47%) as biocatalysts. This observation supports the hypothesis that the decrease in conversion is directly linked to structural changes in the conformation of BCL, which are induced by interactions with the immobilization support, as shown in simulations and FTIR analyses.

In this way, productivity was also estimated based on the performance of the biocatalyst, as shown in Figure 8B. Considering the protein loading of BCL (9.02 mg_protein_·g_enzyme_^–1^) and SiO_2_–BCL (10.63 mg_protein_·g_enzyme_^–1^), when the experimental screening was carried out, it was found that the productivity values in the esterification reaction BCL (150.82 μmol·h^–1^·mg^–1^) > SiO_2_–BCL (28.10 μmol·h^–1^·mg^–1^). These data indicated that an increase in α-helix content in SiO_2_–BCL was inversely correlated with catalytic productivity; in other words, the higher the α-helix content, the lower the productivity.

## Conclusions

The present study demonstrates the potential of computer simulations to predict efficiency and behavior at the molecular level in the enzyme-support complex. Thus, BCL samples prepared using the sol–gel technique (SiO_2_–BCL) exhibited many changes in their catalytic activities and structural properties. Subsequently, the docking simulations showed that only the functional group Q^3^ interacted with the amino acid of the catalytic triad (ser87), and an energy of about −9.4 kcal·mol^–1^ was required for the interaction, which may have restricted the access of the substrate to the catalytic triad.

In conjunction with the FTIR analysis, it can be shown that the synthesis process of the biocatalyst changes in the secondary structure of BCL, which partially hindered the access of the substrate to the active site. Experimental findings confirmed that esterification efficiency depends on the enzyme’s optimal conformation for the stability of the biocatalyst in catalytic activity. In this way, combining available techniques for characterizing interactions can provide a fundamental understanding of the mutual interactions at the protein-silica interface, which has significant implications for further research.

## Supplementary Material



## Notes

FTIR spectra of free BCL, pure SiO_2_, and SiO_2_–BCL (Figure S1); two-dimensional molecular docking simulations showing the spatial positioning and interactions of BCL immobilized on silica (Figure S2) (PDF)
