# Supplementary material for: Enzyme–Support Interactions of Burkholderia cepacia Lipase Immobilized on Silica Using Molecular Docking and Multitechnique Characterization
Source: ACS Appl Mater Interfaces. 2025 Sep 15;17(38):54200–13. doi: 10.1021/acsami.5c11931 (PMC12464904; doi:10.1021/acsami.5c11931)
Supplement: Supplementary file 1 [file am5c11931_si_001.pdf]

SUPPORTING INFORMATION

Enzyme–support interactions of *Burkholderia cepacia* lipase immobilized on silica using molecular docking and multitechnique characterization

César A. Rodrigues[1], Jefferson C. B. Santos[1], Milson S. Barbosa[3], Nayára B. Carvalho[1] Meirielly S. Jesus[4], Ranyere L. Souza[1,2], Álvaro S. Lima[6], Matheus M. Pereira[5], Cleide M. F. Soares[1,2,4*].

[1] Universidade Tiradentes, Av. Murilo Dantas 300, Farolândia, 49032-490, Aracaju, SE, Brazil

[2] Instituto de Tecnologia e Pesquisa, Av. Murilo Dantas 300, Prédio do ITP, Farolândia, 49032-490, Aracaju, SE, Brazil

[3] Instituto Federal de Educação, Ciência e Tecnologia de Minas Gerais (IFMG) - Campus Governador Valadares, 35057-760, Governador Valadares, MG, Brazil

[4] CISAS - Center for Research and Development in Agrifood Systems and Sustainability, Instituto Politécnico de Viana do Castelo, Rua da Escola Industrial e Comercial Nun'Alvares 34, 4900-347, Viana do Castelo, Portugal

[5] University of Coimbra, CERES, Department of Chemical Engineering, Rua Sílvio Lima, Pólo II – Pinal de Marrocos, 3030-760 Coimbra, Portugal

[6] Departamento de Engenharia Química, UFBA, Universidade Federal da Bahia, Rua Aristides Novis 2, Federação, Salvador, BA, Brazil

Additional FTIR Spectra

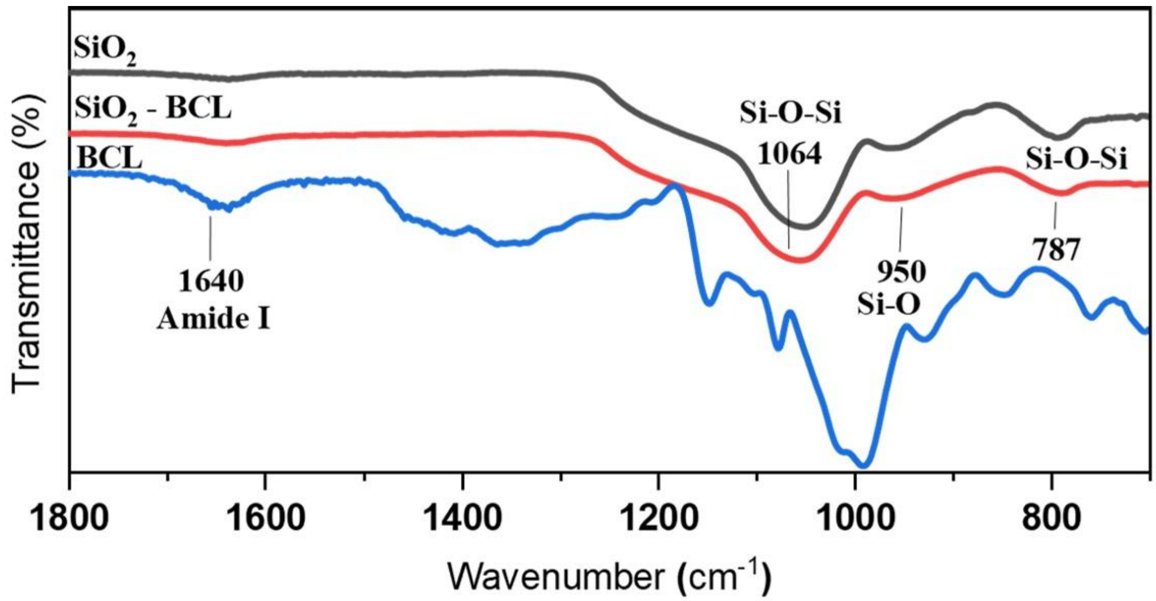

Figure S1. shows the FTIR spectra of free BCL, pure SiO_2, and SiO_2–BCL. Characteristic bands were highlighted to support structural changes discussed in the main text.

Additional Molecular Docking simulations 2D

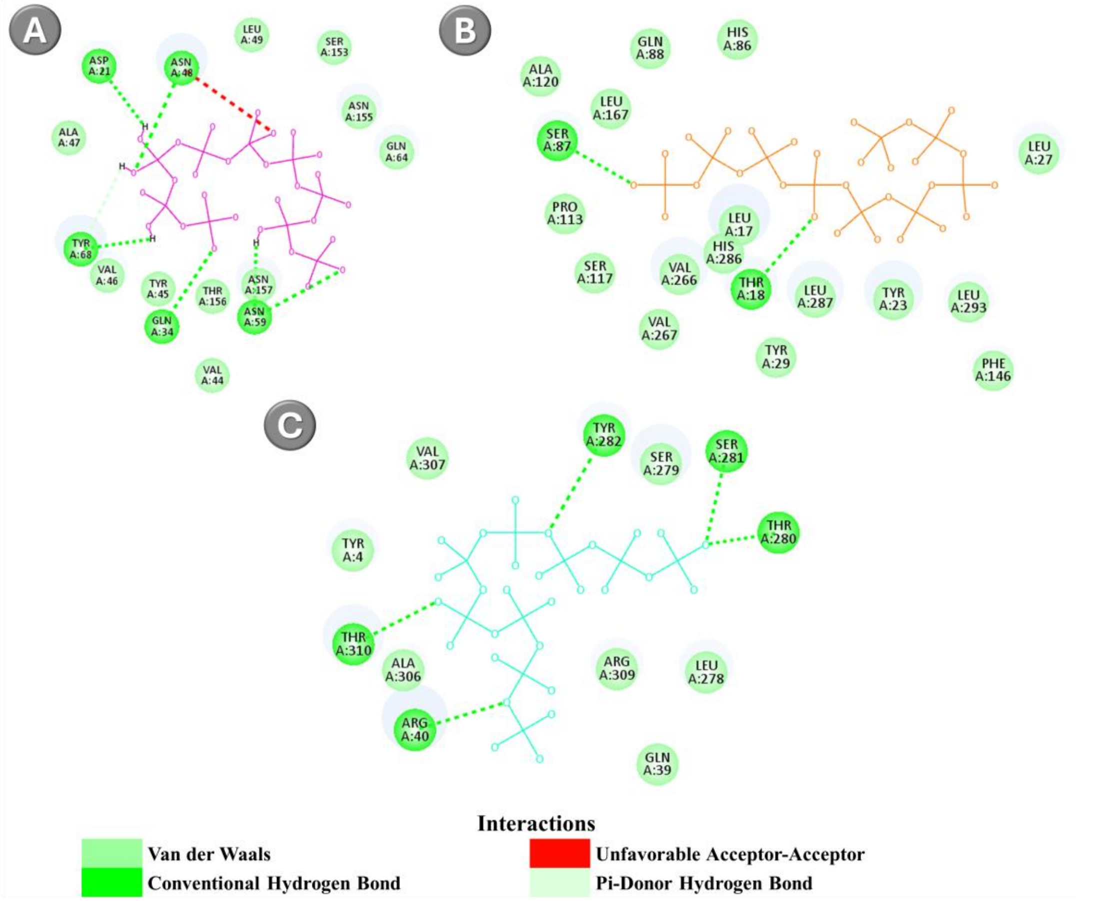

Figure S2. The two-dimensional structure of *Burkholderia cepacia* lipase (BCL, PDB ID: 3LIP) immobilized on the silica surface, (A) Q^2 (purple), (B) Q^3 (orange), and (C) Q^4 (green) representing the spatial positioning of access active site of the biocatalyst immobilized, and individual interactions of functional groups of silica with amino acids of the lipase predicted by AutoDock Vina.
